# In-Stent Restenosis After Seven Years of Endovascular Surgery in a Case of Traumatic Popliteal Artery Pseudoaneurysm Following Total Knee Arthroplasty: A Case Report With Eight Years of Follow-Up

**DOI:** 10.7759/cureus.90640

**Published:** 2025-08-21

**Authors:** Prince Agrawal, Vipin Mohan, Balu C Babu, Druvan Shaji

**Affiliations:** 1 Orthopaedics, Amrita Institute of Medical Sciences and Research Centre, Kochi, IND; 2 Orthopaedics and Traumatology, Amrita Institute of Medical Sciences and Research Centre, Kochi, IND

**Keywords:** endovascular stenting, outcome, popliteal artery, total knee arthroplasty, vascular injury

## Abstract

Popliteal artery aneurysm is a rare but dangerous complication that can occur after total knee arthroplasty (TKA). Early identification and prompt treatment are necessary for a favorable outcome. We can observe a shift toward endoscopic surgical procedures, replacing open surgical procedures to address this treacherous complication. We report a case of missed popliteal artery pseudoaneurysm following an uneventful bilateral TKA, which was initially treated with evacuation of hematoma and compression bandaging by the primary surgeon. Upon presentation, the patient was treated with embolization and antegrade endovascular stenting. The patient was maintaining a good functional outcome until seven years of surgery, following which the patient developed claudication of the same limb. He was diagnosed with in-stent restenosis (ISR) of the popliteal covered stent without chronic limb-threatening ischemia (CLTI) and was managed with oral medications. Patient is comfortable with the oral medications at his eight-year follow-up.

## Introduction

With a documented incidence ranging from 0.03% to 0.17%, trauma to the popliteal artery or its branches is a potential but rare complication of total knee arthroplasty (TKA) [1-3]. The geniculate arteries, popliteal arteries, superficial femoral arteries, and anterior tibial arteries (ATAs) were the most commonly damaged vessels, according to a long-term systematic study and meta-analysis of vascular injuries in TKA [4]. Due to the proximity of the popliteal artery to the knee joint, during retraction or while taking bone cuts, the popliteal artery is susceptible to intraoperative trauma. One of the sequelae of popliteal artery trauma is a popliteal artery pseudoaneurysm [5]. A pseudoaneurysm is a contained hematoma communicating with the artery, unlike a true aneurysm.

We present a case of a popliteal artery pseudoaneurysm in the left lower limb, a complication following an otherwise uneventful bilateral TKA. The initial treatment by the primary surgeon involved hematoma evacuation and compression bandaging, without addressing the pseudoaneurysm. Upon presentation to our institution, the patient was successfully treated with antegrade endovascular stenting of the left popliteal artery pseudoaneurysm using a covered stent. The patient maintained a good functional outcome for seven years post-surgery, at which point he developed claudication in the left lower limb. The claudication was suspected to be due to in-stent restenosis (ISR) of the popliteal covered stent without chronic limb-threatening ischemia (CLTI), and the patient is currently being managed with oral medications.

## Case presentation

A 71-year-old gentleman from South India presented to our hospital after six weeks of bilateral TKA done at an outside facility for primary osteoarthritis of the knee, with complaints of swelling and pain over the left knee associated with difficulty in walking. These symptoms were noticed two days after the surgery. After evaluation, he underwent arthrotomy and evacuation of hematoma of the same knee two weeks following the TKA, but the knee swelling had recurred.

The patient was brought to our hospital six weeks after bilateral TKA, and upon clinical examination of his left knee, effusion was present, and a pulsatile mass was palpable in the popliteal fossa. There were no signs of vascular claudication; both dorsalis pedis and posterior tibial arteries (PTAs) were palpable equally on both limbs without any neurological deficits.

**Figure 1:**
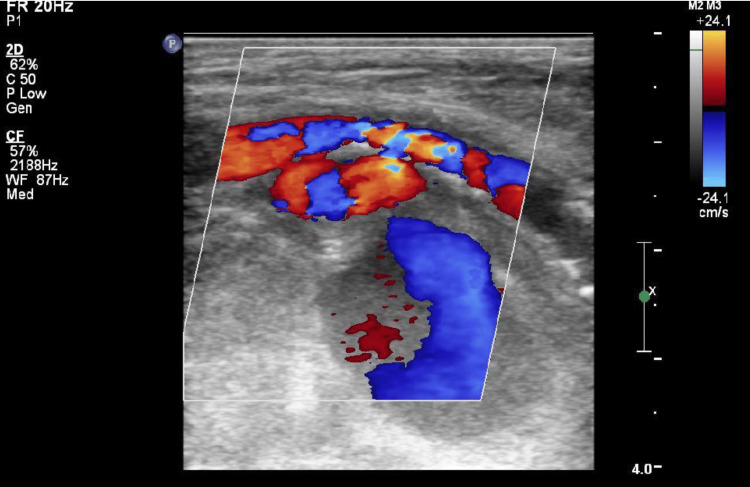
Duplex scan of left popliteal fossa showing a pseudoaneurysm with classical flow characteristics.

**Figure 2:**
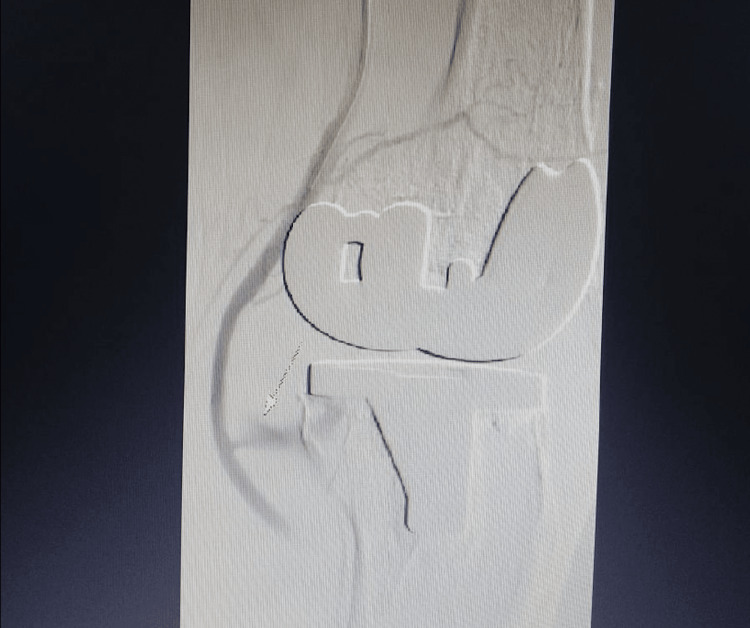
Antegrade angiogram revealing a large popliteal artery pseudoaneurysm (part 1).

**Figure 3:**
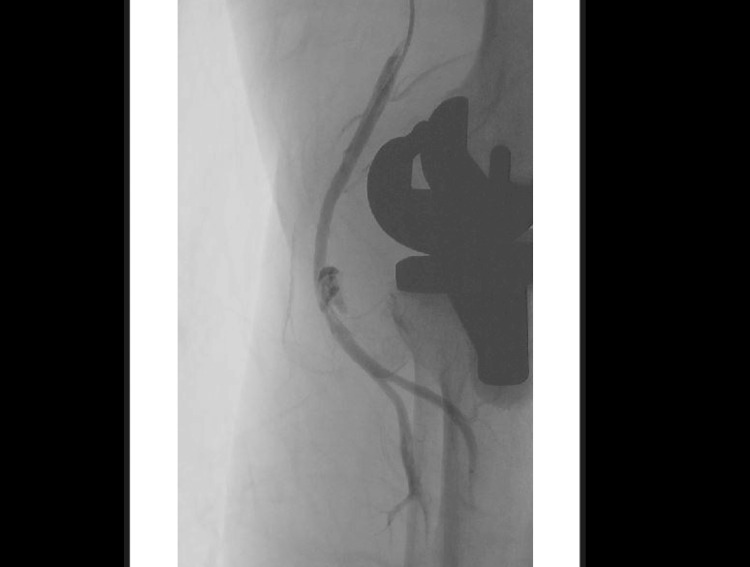
Antegrade angiogram revealing a large popliteal artery pseudoaneurysm (part 2).

An angiogram with ultrasound correlation was performed, and it revealed a rent of 2 mm in size on the anterior wall of the left popliteal artery just below the level of the knee joint with a large pseudoaneurysm measuring about 6 × 3.2 cm in the infra-popliteal level, slightly compressing and displacing the popliteal artery posteriorly (Figures 1-3).

**Figure 4:**
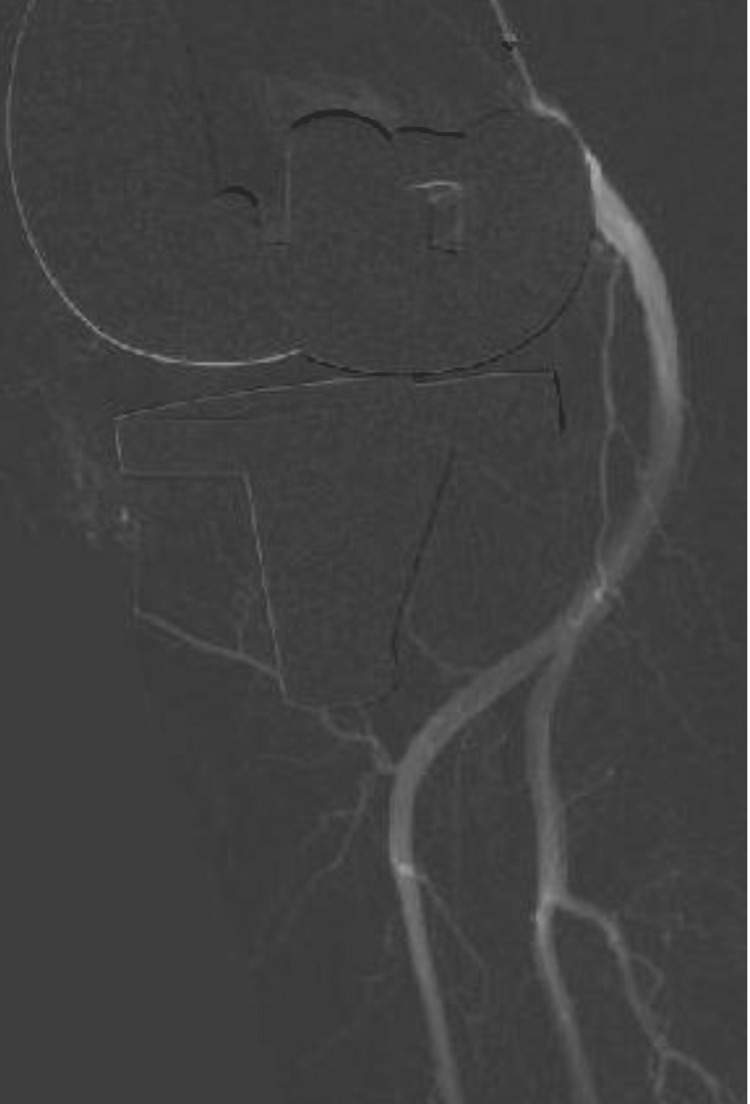
Completion angiogram post-thrombin injection, revealing sealed pseudoaneurysm.

The relevant laboratory values were as follows: international normalized ratio (INR): 0.9; platelet count: 230,000/μL; bleeding time (BT): two minutes; and clotting time (CT): nine minutes. The patient underwent embolization of a pseudoaneurysm of the left popliteal artery. The procedure involved percutaneous thrombin injection, guided by ultrasound sonography (USG), with a 5-mm balloon placed across the ostium of the pseudoaneurysm (Figure 4).

A post-embolization check injection demonstrated thrombus formation within the aneurysm sac and good flow in the popliteal artery. A balloon-mounted covered stent called LifeStream™ 5 × 37 mm (Beckton Dickinson, Franklin Lakes, NJ) was then used to cover a tiny rent in the left popliteal artery. Knee immobilization was maintained for two weeks, followed by the initiation of knee range of motion exercises, quadriceps strengthening, and weight-bearing activities.

He was reviewed periodically with clinical examination and duplex scans at three months, six months, 12 months, and then annually. Duplex scan at six years follow-up showed good flow in the ATA and PTA. The graft appeared patent with good flow noted within without any evidence of thrombus within the stent.

**Figure 5:**
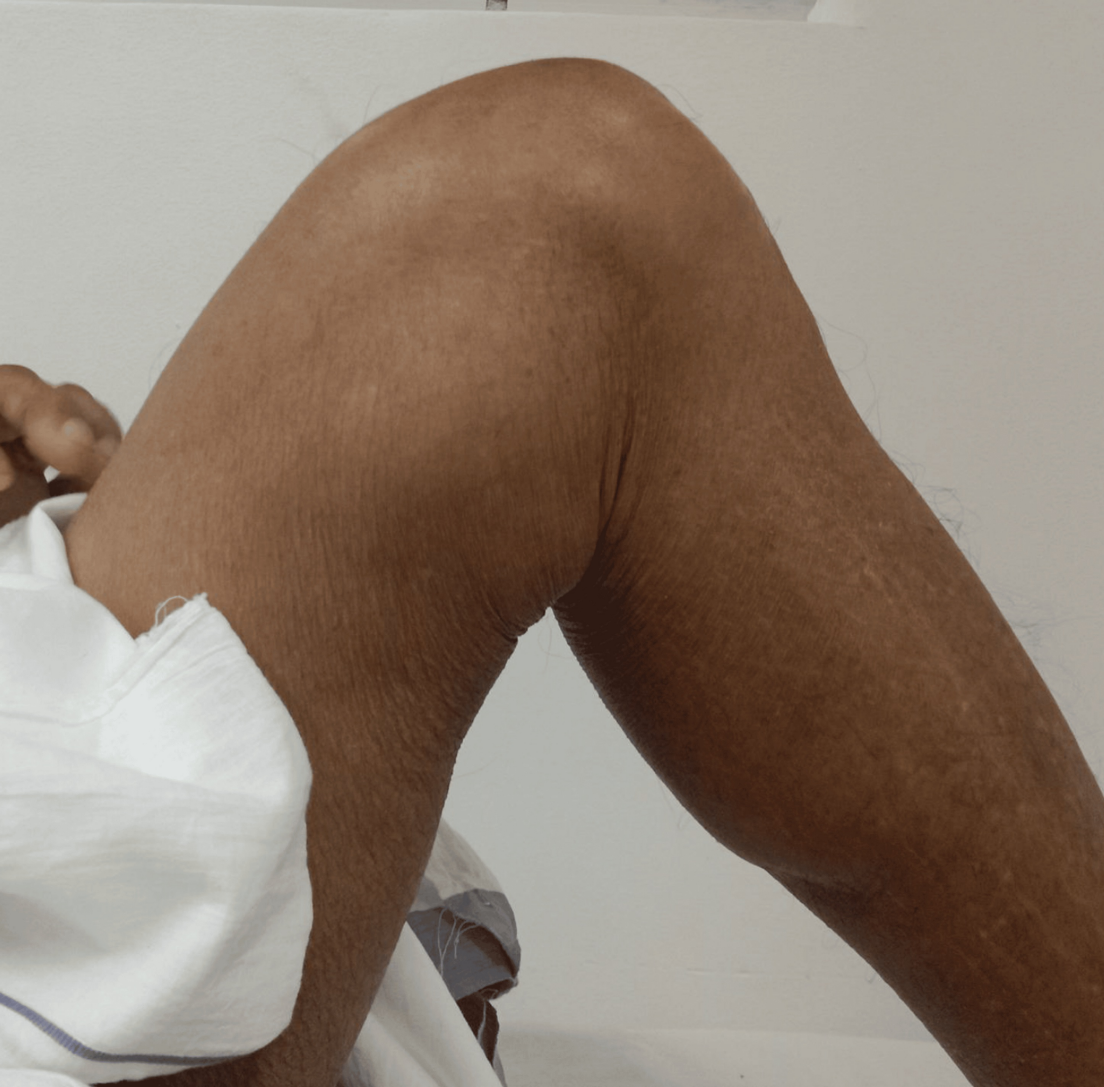
Clinical images at eight years postoperatively.

After seven years of surgery, he was walking independently without any vascular claudication and was maintaining good function (Figure 5).

**Figure 6:**
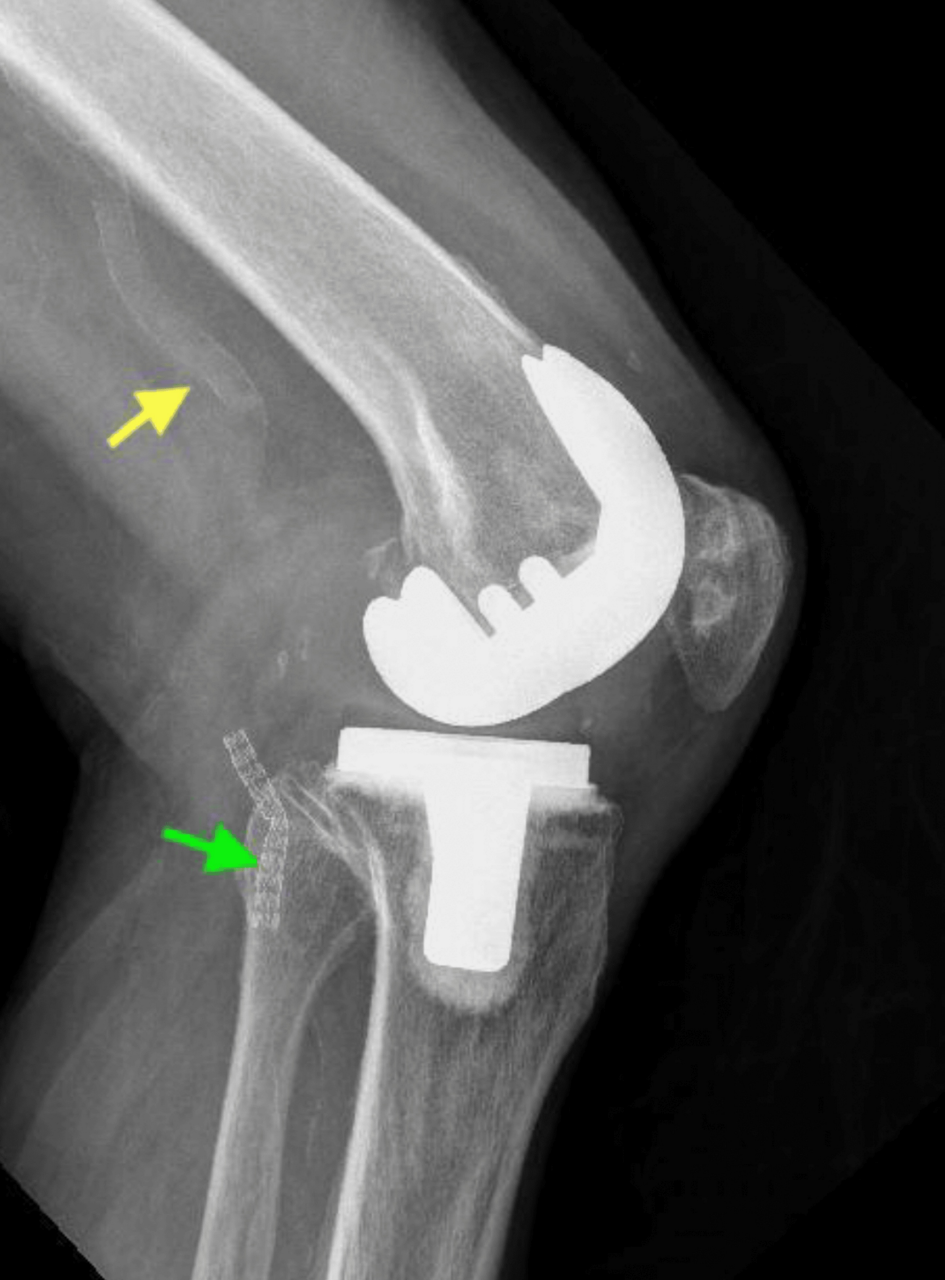
Lateral radiograph eight years post-op, revealing calcified popliteal artery (yellow arrow) and the covered endovascular stent in position (green arrow).

The clinical Knee Society Score was 88/100 on the left side and 95/100 on the right side, and the functional Knee Society Score was 90/100 bilaterally. His X-rays also revealed the maintenance of the stent position (Figure 6).

**Figure 7:**
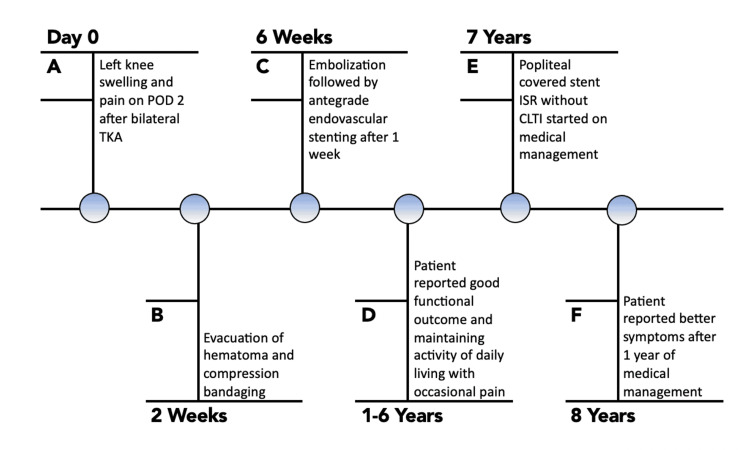
Timeline of the clinical events.

At his seven-year follow-up, the patient complained of pain in the left lower leg in the calf region for the past three months that aggravates on walking and relieves on rest, typical of vascular claudication. Symptoms were mild, not disabling his day-to-day activities. There was no history of rest pain (Figure 7). 

On examination, the temperature of the foot was normal on both sides. Femoral and popliteal pulses were well palpable bilaterally. Left dorsalis pedis and PTAs were not palpable. On further evaluation with an arterial duplex scan, moderate monophasic PTA signals were present on the left side; the quantitative velocity data of which were not recorded, but qualitative monophasic signals suggested moderate stenosis. There were good biphasic PTA signals present on the right side. Patient was suspected to have ISR of the stent with diffusely diseased tibialis with monophasic flow pattern. He was diagnosed with Rutherford 3 (grade I) peripheral artery disease - left below the knee without signs of CLTI. A consultation with a vascular surgeon was sought, and the patient was advised on medical line of management with statins and antiplatelets (Cilostazol 100 mg). He was counselled about his condition and was informed about the importance of being compliant with the treatment. He was also advised to do walking exercises and foot care.

On his latest follow-up, eight years after the initial surgery, his symptoms were better with medications, though he still complained of intermittent difficulties. The patient is managing mobilization independently. Moderate (grade II) pedal Doppler signals were noted, and the patient was advised to continue the same treatment regimen.

## Discussion

According to the literature, the incidence of popliteal artery pseudoaneurysm ranges from 0.002% and 0.037% of TKA cases [3,5], which may present early as a pulsatile mass in the popliteal fossa, calf swelling, or occasionally with features of ischemia present after the initial procedure [6]. The presentation may be late with an insidious onset, ranging from two days to five months postoperatively [7]. Ammori et al. found a median interval of 15 days (range: 7-27 days) [8].

Indirect trauma is the predominant mechanism for pseudoaneurysm formation, encompassing mechanical stretching (such as placement of the retractor behind the tibial plateau) [2], release of the posterior capsule, dislocation of the knee joint for preparation of the posterior femoral condyle and proximal tibia, and thermal injury or compression resulting from cement [1,9].

In this case, intraoperative data are not available as the primary surgery was done elsewhere. However, the pre-operative X-rays of the patient show bilateral tricompartmental osteoarthritis of the knee with varus deformity, with calcified popliteal arteries (Figure 3), which possibly made it more susceptible to a vascular injury, which led to the development of a popliteal pseudoaneurysm.

Vigilance in the clinical suspicion of such a rare complication is difficult during the postoperative period, as a popliteal artery pseudoaneurysm is not a well-known complication to orthopedic surgeons [10]. Once identified, due to the nature of the trauma, only a small percentage of these lesions may be treated locally [3].

The standard of care for the remaining patients is open repair combined with closure of the native artery and above-to-below knee popliteal artery bypass [11]. This can be an unappealing prospect, as it involves harvesting a venous conduit from the contralateral limb and operating in a hostile operative field, thereby exposing a recently placed prosthesis to the risk of potential infection.

A covered stent is placed across the popliteal artery defect as part of endovascular treatment. According to the evidence currently available, endovascular treatment is a durable and safe treatment modality for the management of traumatic popliteal artery pseudoaneurysms with a high success rate [12]. Nonetheless, the popliteal artery's relative mobility raises concern as it increases the risk of stent fracture or device migration. However, follow-up duplex scanning suggests that, with time, the stent graft becomes incorporated into the vessel wall by the six-month follow-up [3].

Another source of concern is the long-term patency of the popliteal artery stent. Although cases of femoropopliteal ISR have been reported in the literature, which were done for other causes, no case of ISR has been reported in a patient with a popliteal artery pseudoaneurysm following TKA.

## Conclusions

Even in an uneventful TKA procedure, one should have a clinical suspicion of a rarer vascular complication, such as a pseudoaneurysm of the popliteal artery. This uncommon complication can have a delayed presentation after being missed in the immediate postoperative period. Along with the routine evaluation of distal pulses, this case demonstrated the importance of the clinical assessment of the popliteal fossa. Therefore, in these dubious situations following TKA, we advise using duplex ultrasound to screen for vascular abnormalities in the popliteal fossa. Endovascular repair using a covered stent is a safe, less invasive, and durable procedure in the management of popliteal artery pseudoaneurysm. This case also shows that with time, ISR is a possible complication that can cause claudication pain, which an orthopedic surgeon should be vigilant about during follow-ups.
